# Immune profiling of NF1-associated tumors reveals histologic subtype distinctions and heterogeneity: implications for immunotherapy

**DOI:** 10.18632/oncotarget.18301

**Published:** 2017-05-30

**Authors:** Kellie B. Haworth, Michael A. Arnold, Christopher R. Pierson, Kwangmin Choi, Nicholas D. Yeager, Nancy Ratner, Ryan D. Roberts, Jonathan L. Finlay, Timothy P. Cripe

**Affiliations:** ^1^ Division of Hematology, Oncology, Blood and Marrow Transplant, Department of Pediatrics, Nationwide Children’s Hospital, Columbus, Ohio, USA; ^2^ Center for Childhood Cancer and Blood Diseases, The Research Institute, Nationwide Children’s Hospital, Columbus, Ohio, USA; ^3^ Division of Anatomic Pathology, Department of Pathology and Laboratory Medicine, Nationwide Children’s Hospital, Columbus, Ohio, USA; ^4^ Department of Pathology, The Ohio State University College of Medicine, Columbus, Ohio, USA; ^5^ Division of Anatomy, Department of Biomedical Education and Anatomy, The Ohio State University College of Medicine, Columbus, Ohio, USA; ^6^ Division of Experimental Hematology and Cancer Biology, Cincinnati Children’s Hospital Medical Center, Cincinnati, Ohio, USA

**Keywords:** NF1, MPNST, neurofibromas, immunotherapy, immunophenotype

## Abstract

Successful treatment of neurofibromatosis type 1 (NF1)-associated tumors poses a significant clinical challenge. While the primary underlying genetic defect driving RAS signaling is well described, recent evidence suggests immune dysfunction contributes to tumor pathogenesis and malignant transformation. As immunologic characterizations, prognostic and predictive of immunotherapeutic clinical response in other cancers, are not fully described for benign and malignant NF1-related tumors, we sought to define their immunologic profiles. We determined the expression of human leukocyte antigen (HLA)-A/-B/-C, β-2-microglobulin (B2M), and T cell inhibitory ligands PD-L1 and CTLA-4 by microarray gene analysis and flow cytometry. We examined HLA-A/-B/-C, B2M, and PD-L1 expression on thirty-six NF1-associated tumor samples by immunohistochemistry, and correlated these with tumoral CD4^+^, CD8^+^, FOXP3^+^, CD56^+^, and CD45RO^+^ lymphocytic infiltrates. We evaluated several tumors from a single patient, observing trends of increasing immunogenicity over time, even with disease progression. We observed similarly immunogenic profiles for malignant peripheral nerve sheath tumors (MPNST) and nodular and plexiform neurofibromas, contrasting with diffuse neurofibromas. These studies suggest that while immunotherapies may offer some benefit for MPNST and nodular and plexiform neurofibromas, tumor heterogeneity might pose a significant clinical challenge to this novel therapeutic approach.

## INTRODUCTION

Neurofibromatosis Type I (NF1) is an autosomal dominant disorder affecting approximately one in every 3500 people [1]. The syndrome results from a mutation in the NF1 gene encoding neurofibromin, a protein ubiquitously expressed in tissues throughout the body [2] and involved in the RAS signaling pathway [3]. Diagnostic criteria developed by the National Institutes of Health illustrate a myriad of clinical features related to the disease [4], with nervous tissue overgrowth as a universal feature. Virtually all patients with NF1 suffer the development of benign peripheral nerve sheath neurofibromas[5], believed to arise from aberrant Schwann cells [6, 7].

Several benign neurofibroma subtypes have been described based upon location (dermal and intra-neural), macroscopic growth pattern (localized, diffuse, or plexiform), and histopathologic appearance (nodular or diffuse) [8, 9]. Though localized and dermal diffuse tumors arise from distal nerve ends and are not known to undergo malignant transformation, they are problematic for aesthetic reasons. Diffuse intra-neural tumors may cause pain and potentially severe neurologic deficits [2]. Nodular neurofibromas, which arise from nerve roots, often cause morbidity related to their growth pattern within organs [2]. The plexiform variant, involving long segments of multiple nerves, are found in any tissue layer where nerves are present, and represent the highest morbidity for NF1 patients due to tissue compression. Even more concerning is the 20% likelihood of evolution into a malignant peripheral nerve sheath tumor (MPNST)[2], which confers only 28% 5-year overall survival [10].

The development and progression of NF1-associated tumors are primarily linked to aberrant RAS signaling. Recent evidence suggests immune system dysfunction may also play a role. RAS signaling plays a pivotal role in myelopoiesis, and its constitutive activation upon NF1 loss is associated with myeloid progenitor cell hyper-responsiveness to growth factors [3]. Plexiform neurofibroma tumorigenesis in some mouse models is dependent upon NF1 haploinsufficiency within the hematopoietic compartment [11]. The promotion of tumor formation and growth by mast cell-induced inflammation in these models suggests an immunologic microenvironmental contribution to tumoral pathogenesis [12].

Low expression of genes critical for immunity may provide immune escape and avoidance of cytotoxic T lymphocyte (CTL) engagement in the context of malignant transformation to MPNST. Such genes, including the human leukocyte antigen (HLA)-encoding major histocompatibility complex (MHC) class I and II and the transcription factor MHC II transactivator (MHC2TA), the transporter associated with antigen processing (TAP1), and the related chaperone CD74, have reduced expression in NF1-associated tumors compared to normal human Schwann cells [13]. In contrast, the expression of the necessary MHC Class I-associated protein β-2-microglobulin (B2M) is up-regulated among neurofibromas [14]. Though a recent study characterizing tumoral expression of the ligand for programmed cell death protein-1 (PD-1, or CD279), PD-L1, in MPNST tumors reported no correlation with patient outcomes[15], PD-1-positive lymphocyte infiltration and tumoral PD-L1 expression have previously been reported to correlate with decreased overall and event-free survival rates for soft tissue sarcomas including MPNST [16]. Therefore, restoration of appropriate immune function may be important for the prevention of tumor growth and progression.

Here we sought to define the immunologic profiles of both benign and malignant nerve tumors associated with NF1. We characterized the expression of the immunologic markers MHC Class I and B2M and the T cell inhibitory ligands PD-L1 and cytotoxic T lymphocyte antigen-4 (CTLA-4) by gene analysis on tumor and cell line microarrays and determined cell line protein expression of these markers by flow cytometry. We examined HLA I, B2M, and PD-L1 expression on pediatric and young adult tumor samples by immunohistochemistry.

The number of tumor-infiltrating CTLs directly correlates with prognosis in various adult cancer types. In fact, an “immunoscore” based upon CTL (CD3^+^CD8^+^) and T-memory (CD3^+^CD45RO^+^) infiltrates has been proposed to augment traditional tumor, node, metastasis staging [17]. The immunoscore was found to be the strongest prognostic factor for disease-free, disease-specific, and overall survival for colorectal cancers [17]. We thus also characterized tumoral immunologic cellular infiltrates, including CD4^+^, CD8^+^ (CTL), FOXP3^+^ (suppressive), CD45RO^+^ (memory), and CD56^+^ (NK) lymphocytes, and correlated these with our immunohistochemical staining scores.

Our data suggest that the immunologic profiling of NF1-associated tumors may aid in the selection of immune-based therapeutic strategies. Interestingly, we found that benign neurofibromas are distinct in their immunologic profiles and that specific subtypes may also benefit from immunotherapeutic approaches.

## RESULTS

### NF1-associated tumors and tumor-related Schwann cells display decreased HLA I and B2M and variable T cell inhibitory ligand gene expression compared to normal human Schwann cells

**Figure 1:**
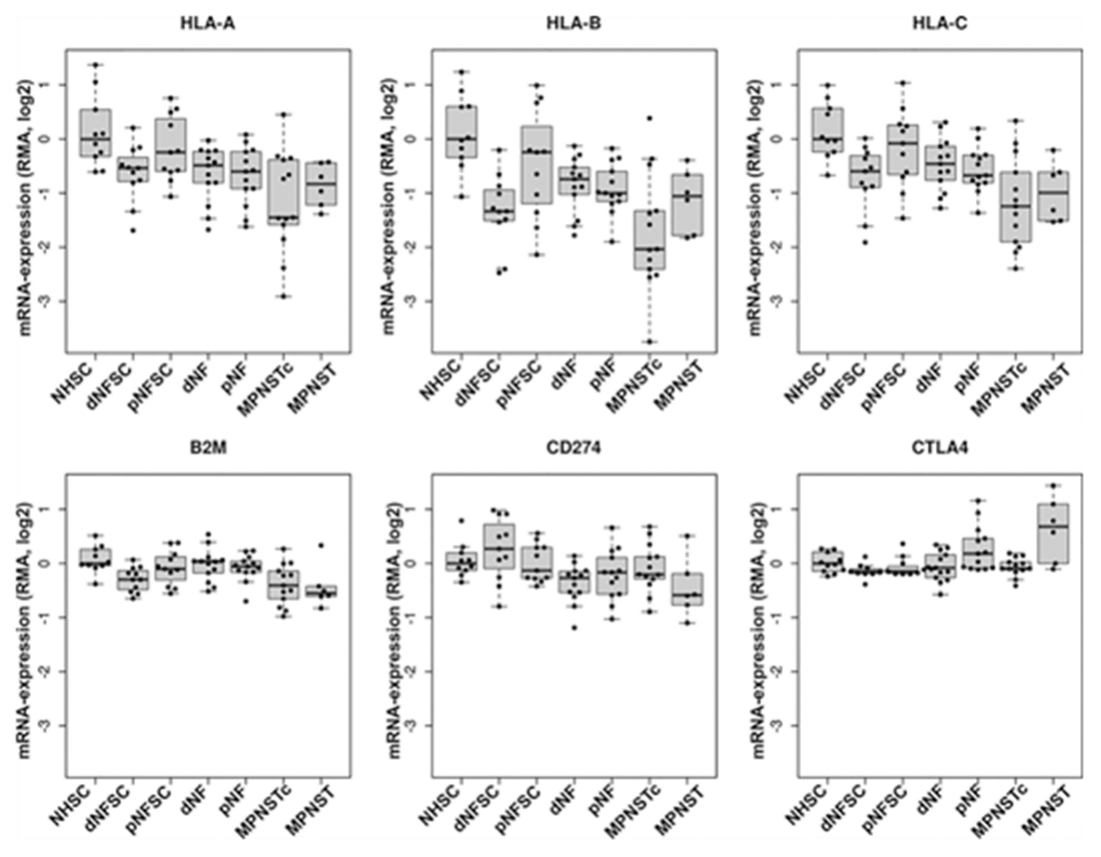
Expression of immunologic markers in NF1-associated tumors by gene microarray The microarray represents 13 dermal neurofibromas, 13 plexiform neurofibromas, 6 MPNST tumors, and 13 MPNST cell lines normalized against 10 samples of normal human Schwann cells (GSE14038). Top: HLA-A, HLA-B, and HLA-C (MHC Class I); bottom left: B2M; bottom middle: CD274 (PD-L1); bottom right: CTLA-4. The mean of all samples within tumor subtype with standard error of mean are shown. NHSC= normal human Schwann cell; dNFSC= dermal neurofibroma Schwann cell; pNFSC= plexiform neurofibroma Schwann cell; dNF= dermal neurofibroma; pNF= plexiform neurofibroma; MPNSTc= malignant peripheral nerve sheath tumor cell; MPNST= malignant peripheral nerve sheath tumor.

As an initial assessment of immunologic molecule expression in NF1-associated tumors, we queried a microarray database for genes of interest (HLA-A/B/C, B2M, PD-L1, and CTLA-4) (Figure 1). We found that HLA-A, -B, and -C were down-regulated in both benign and malignant NF1-associated tumors and tumor-associated Schwann cells compared to normal human Schwann cells, with the lowest expression noted in MPNST cell lines. B2M gene expression was lowest in malignant MPNST samples, with slightly lower expression in tumors than in cell lines. Benign NF1-associated tumors did not display significantly different B2M expression compared with normal human Schwann cells. Expression of the gene encoding the CTL inhibitory ligand CD274 (PD-L1) was lowest in MPNST and dermal neurofibroma tumors. Dermal neurofibroma Schwann cells, however, displayed a range of PD-L1 gene expression, with an average overall higher than that of normal human Schwann cells. In contrast, gene expression of the CTL inhibitory ligand CTLA-4 was notably up-regulated in the majority of MPNST tumors and several plexiform neurofibroma tumor samples. CTLA-4 was not elevated in MPNST cell lines or tumor-associated Schwann cells compared with normal human Schwann cells.

### MPNST cell lines express HLA-A/B/C and B2M, variably exhibit PD-L1, and do not express CTLA-4 by flow cytometry

We selected two MPNST cell lines, one a spontaneous non-NF1-associated tumor and the other an NF1-associated tumor-derived cell line, to perform flow cytometric analysis of immunologic molecule expression (Supplementary Figure 1). We detected HLA-A/B/C and B2M in both cell lines. PD-L1 expression was high in the spontaneous model STS-26T, but only very slightly expressed in the NF1-associated model S462TY. We did not detect significant CTLA-4 protein expression in either MPNST cell line.

### NF1-associated tumors exhibit wide ranges of HLA-A/B/C, B2M, and PD-L1 expression by immunohistochemical staining, revealing differences between histologic subtypes

**Figure 2:**
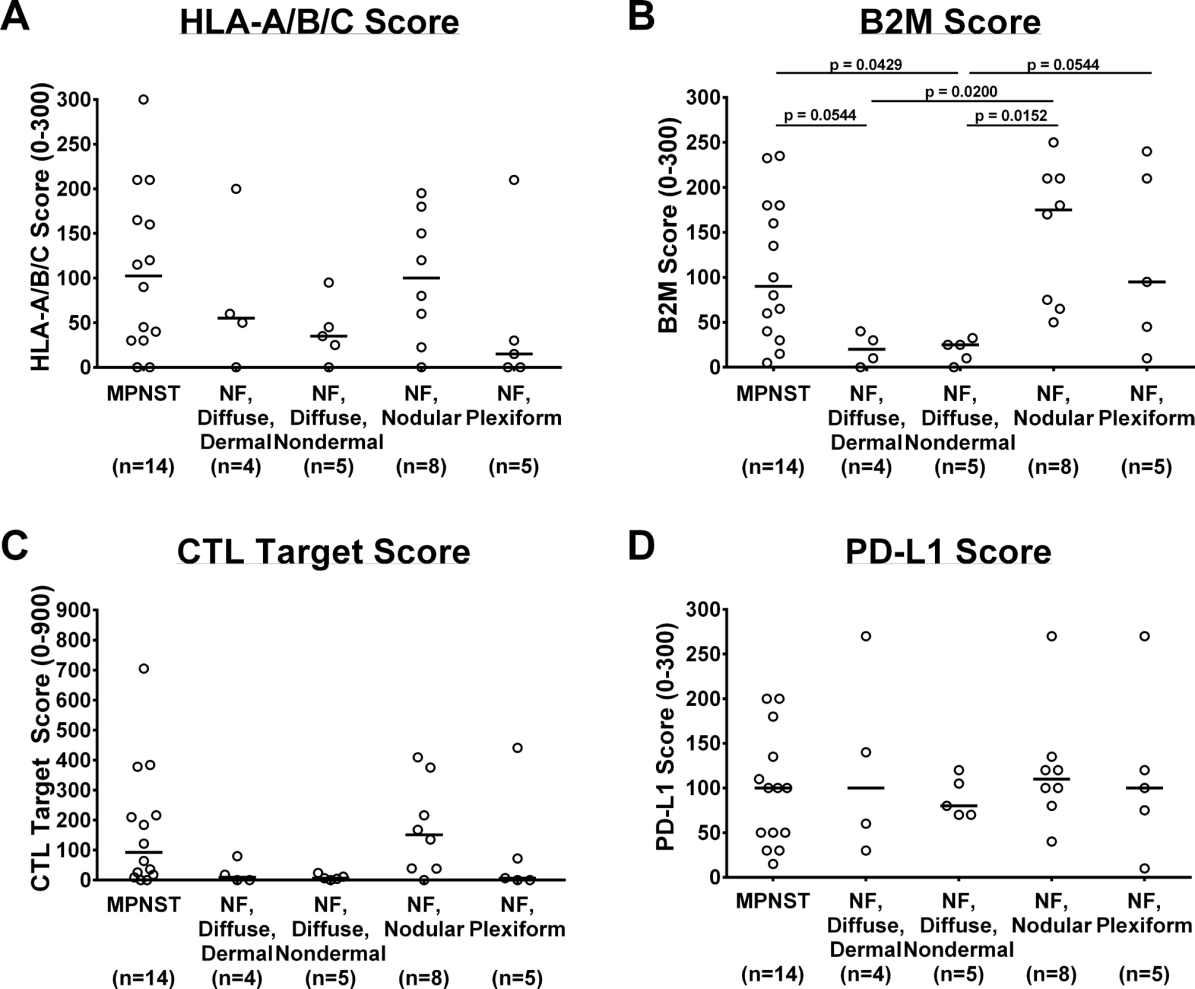
*In vivo* protein expression of immunologic markers in NF1-associated tumors by immunohistochemistry **(A)** HLA-A/B/C, range 0 to 300; **(B)** B2M, range 0 to 300; **(C)** CTL Target Scores, range 0 to 900; and **(D)** PD-L1, range 0 to 300. Each data point represents average of 3 technical replicates for that sample. Number of samples per tumor subtype listed in parentheses below tumor subtype labels. Median of all samples within tumor subtype represented by bar. Tumor subtype scores were compared using Kruskal-Wallis non-parametric testing with the two-step method of Benjamini, Krieger, and Yekutieli post-hoc pairwise comparison to control for a false discovery rate of 0.05 with GraphPad Prism 7 software. Significant results (defined as adjusted p ≤ 0.05) indicated on graphs. CTL= cytotoxic T lymphocyte; MPNST= malignant peripheral nerve sheath tumor; NF= neurofibroma.

To assess tumors in the context of their microenvironment, we performed immunohistochemical staining for immunologic markers of interest (HLA-A/B/C, B2M, and PD-L1) on benign (histologies depicted in Supplementary Figure 2) and malignant tumors from NF1 patients and scored their staining intensity and percentage as previously described [17]. Benign and malignant tumors alike demonstrated wide ranges of HLA-A/B/C staining scores within their individual subtype, indicating substantial heterogeneity among tumors of the same histologic type (Figure 2A). To determine if time from diagnosis or therapeutic interventions contribute to the observed heterogeneity, we compared MPNST samples based on their status as diagnostic versus recurrent or autopsy samples (Supplementary Figure 3A-3D). No clear trend emerged for any of the immunologic markers we examined. In general, MPNST and nodular histology benign tumors displayed higher average HLA-A/B/C staining scores, while diffuse and plexiform neurofibromas exhibited lower average scores (Figure 2A). Benign neurofibromas with nodular histology demonstrated the highest average of B2M staining scores of all samples tested, while the diffuse neurofibromas exhibited the lowest average B2M scores (Figure 2B). Taking both HLA-A/B/C and B2M into account, the average calculated CTL Target Scores were similar amongst MPNST and nodular and plexiform neurofibromas, though they again exhibited extensive score variability within each histologic category. In contrast, the diffuse neurofibromas displayed less variability, with most CTL Target Scores close to zero (Figure 2C).

To further evaluate the potential for CTL inhibition, we assessed tumoral PD-L1 expression. The average PD-L1 staining scores did not vary significantly amongst the individual tumor subtypes (Figure 2D). All tumor histologies consistently showed broad ranges of PD-L1 scores within their respective group.

### Immune cellular infiltrates in NF1-associated tumors vary by tumor histology

**Figure 3:**
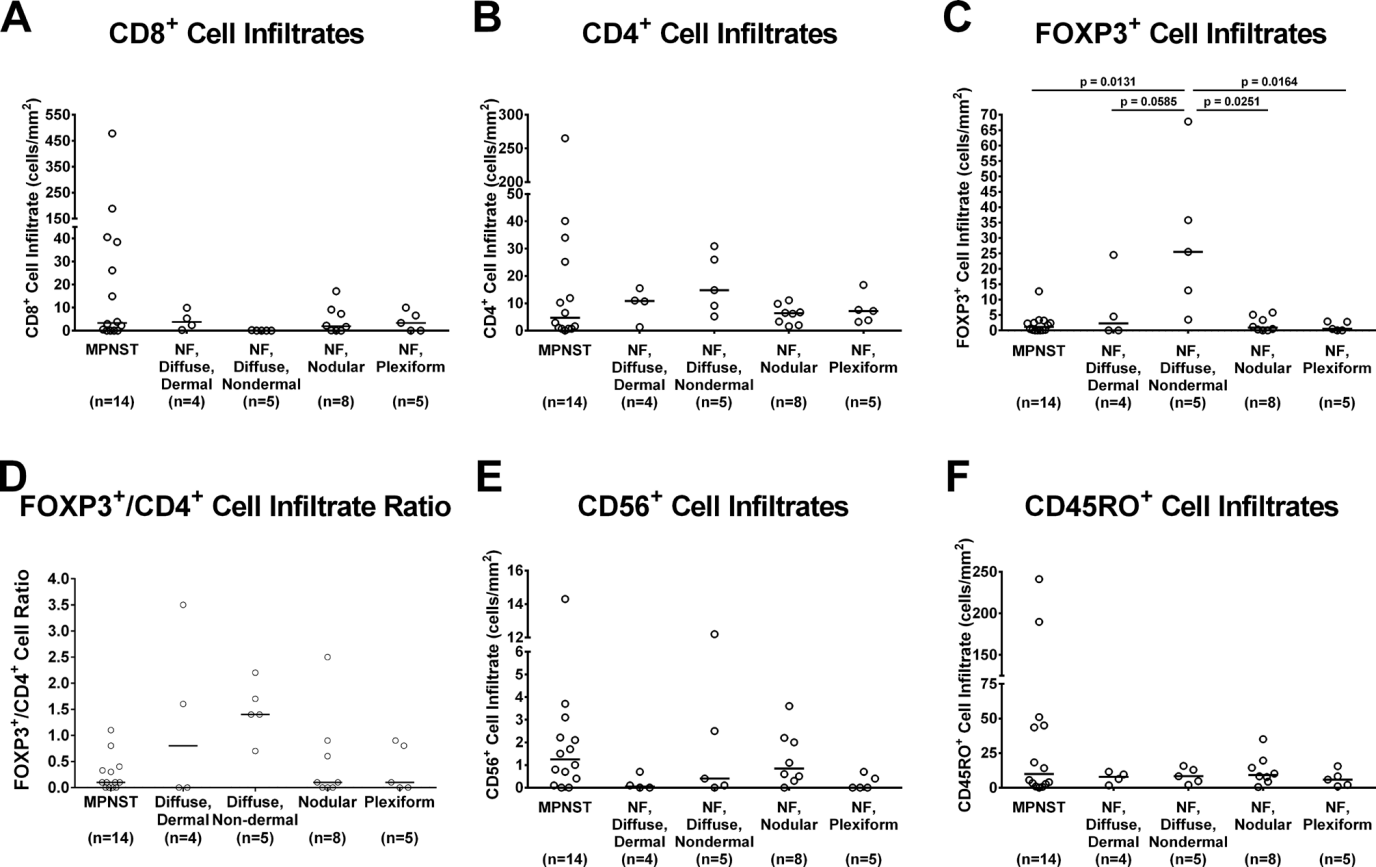
*In vivo* immune cellular infiltrates in NF1-associated patient tumor samples by immunohistochemistry **(A)** CD8^+^ cellular infiltrates; **(B)** CD4^+^ cellular infiltrates; **(C)** FOXP3^+^ cellular infiltrates; **(D)** FOXP3^+^/CD4^+^ cellular infiltrate ratio; **(E)** CD56^+^cellular infiltrates; and **(F)** CD45RO^+^ cellular infiltrates. Each data point represents average of 3 technical replicates for that sample. Number of samples per tumor subtype is listed in parentheses below tumor subtype labels. The median of all samples within each tumor subtype is represented by the bar. Comparisons of cellular infiltrates between each tumor subtype were performed using Kruskal-Wallis non-parametric testing with the two-step method of Benjamini, Krieger, and Yekutieli post-hoc pairwise comparison to control for a false discovery rate of 0.05 with GraphPad Prism 7 software. Significant results (adjusted p ≤ 0.05) are indicated on graphs.

We quantified CD8^+^, CD4+, FOXP3^+^, CD45RO^+^, and CD56^+^ lymphocytes within the tumors (representative staining portrayed in Supplementary Figure 4A-4D). We found that MPNST tumors contain much higher numbers of CD8^+^ (Figure 3A), CD4^+^ (Figure 3B), CD56^+^ (Figure 3E), and CD45RO^+^ (Figure 3F) lymphocytes overall than those observed in the neurofibromas. Among the benign NF1-associated tumors, the nodular, plexiform, and dermal diffuse samples displayed similar trends, with higher median CD8^+^ and lower median CD4^+^ and FOXP3^+^ cellular infiltrates than the non-dermal diffuse histology tumors. The CD4^+^ cells correlated with the FOXP3^+^ cellular infiltrates within the benign tumors. MPNST tumors overall displayed comparatively low FOXP3^+^ cell counts (Figure 3C and 3D), similar to those observed in the nodular and plexiform benign neurofibroma groups. The CD56^+^ cell infiltrate numbers did not significantly vary between MPNST and benign tumors (Figure 3E).

We found no differences in CD4^+^ or CD56^+^ lymphocyte infiltrate numbers in MPNST samples when they were classified according to sample type (diagnostic, recurrent, or autopsy) (Supplementary Figure 5B and 5E). The median numbers of tumor-infiltrating CD8^+^ and CD45RO^+^ lymphocytes were much lower, while median FOXP3^+^ cell infiltrates were higher in recurrent samples compared with diagnostic samples (Supplementary Figure 5A, 5C, and 5F). Autopsy samples showed notable decreases in all cellular infiltrates examined.

### Evaluation of immunologic marker expression and immune cellular infiltrates in NF1-associated tumors from the same patient reveals differences based on tumor histologic subtypes, timing, therapies, and intra-tumoral heterogeneity

We assessed several benign and malignant tumor samples obtained from the same patient at various time points, beginning four years from the patient’s initial MPNST diagnosis at 12 years of age through various biopsies over a period of 13 years, and at autopsy. Sample histologies included benign plexiform, nodular, and dermal diffuse neurofibromas and both low- and high-grade MPNST. The patient had received radiation therapy upon initial MPNST diagnosis, and each sample for the first 12 years after diagnosis occurred without further chemo- or radiotherapeutic interventions. The samples obtained 13 years after diagnosis, however, reflect the patient’s disease after receiving a standard chemotherapeutic course with ifosfamide and doxorubicin, localized palliative irradiation to two lesions, and the addition of biologic agents (bevacizumab, a monoclonal antibody directed against vascular endothelial growth factor (VEGF), and the mammalian target of rapamycin (mTOR) inhibitor everolimus) (see patient case data in Supplementary Table 1).

**Figure 4:**
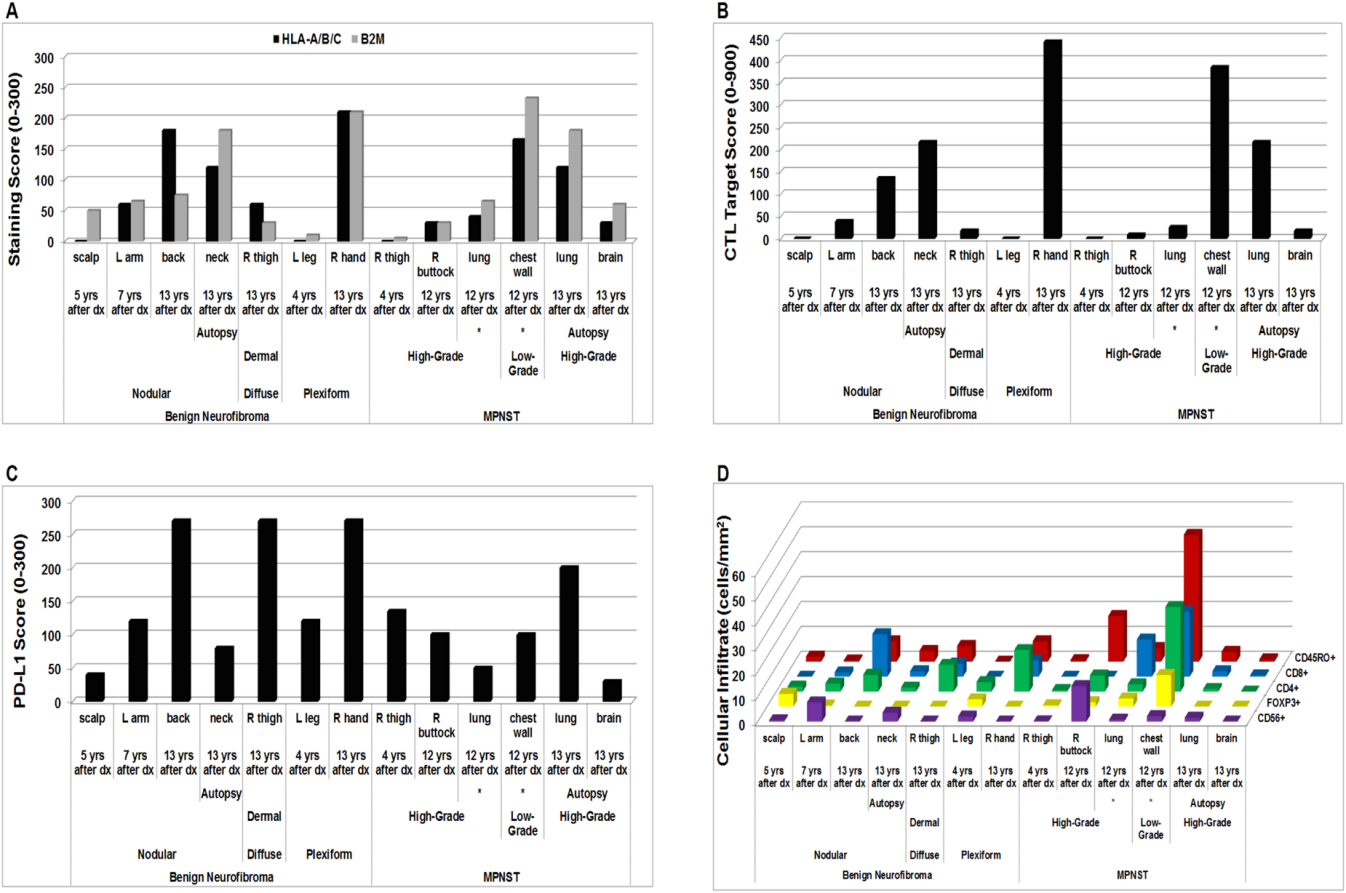
*In vivo* scoring and cellular infiltrates in NF1-associated tumors from the same patient by immunohistochemistry **(A)** HLA-A/B/C and B2M Scoring; **(B)** CTL Target Scores; **(C)** PD-L1 scores; and **(D)** immune cellular infiltrates. Each data point represents average of 3 technical replicates for that sample. Samples obtained from the same tumor mass during the same surgical procedure are indicated by *. Dx = diagnosis; L = left; R = right; yrs = years.

Among the benign tumors sampled from this patient, CTL Target Scores rose over time, reflecting increases in both the HLA-A/B/C and B2M staining scores (Figure 4A). We observed a similar upward trend in CTL Target Scores in the patient’s MPNST samples until autopsy, where CTL Target Scores were notably lower. Interestingly, of the MPNST samples obtained 12 years after original diagnosis, two samples acquired at the same time from the same tumor (chest wall lesion with extension into the lung) showed differences in histologic grade (low- *versus* high-grade) and yielded considerably different staining scores. The low-grade sample displayed higher immunologic marker and CTL Target Scores than the high-grade sample (Figure 4B). PD-L1 staining scores obtained from the patient similarly trended upward in the benign lesions, but reflected an opposite trend to the CTL Target Scores for the malignant tumor samples (Figure 4C).

The intra-tumoral CD8^+^, CD4^+^, and CD45RO^+^ lymphocytic cellular infiltrates displayed upward trends over time for each tumor subtype with the exception of samples obtained at autopsy (Figure 4D). FOXP3^+^ cell numbers decreased over time in the benign samples, but increased in the MPNST tumors (Figure 4D). The CD56^+^ cellular infiltrates did not reveal a distinct pattern among the tumors sampled from the patient (Figure 4D). Of the two MPNST samples obtained at the same time from within the same tumor 12 years after original diagnosis (see Supplementary Figure 6), the low-grade sample exhibited much higher CD8^+^, CD4^+^, FOXP3^+^, CD45RO^+^, and CD56^+^ lymphocytic infiltrates than did the high-grade MPNST sample (Figure 4D).

### Immunologic marker staining scores correlate with cellular infiltrates

**Figure 5:**
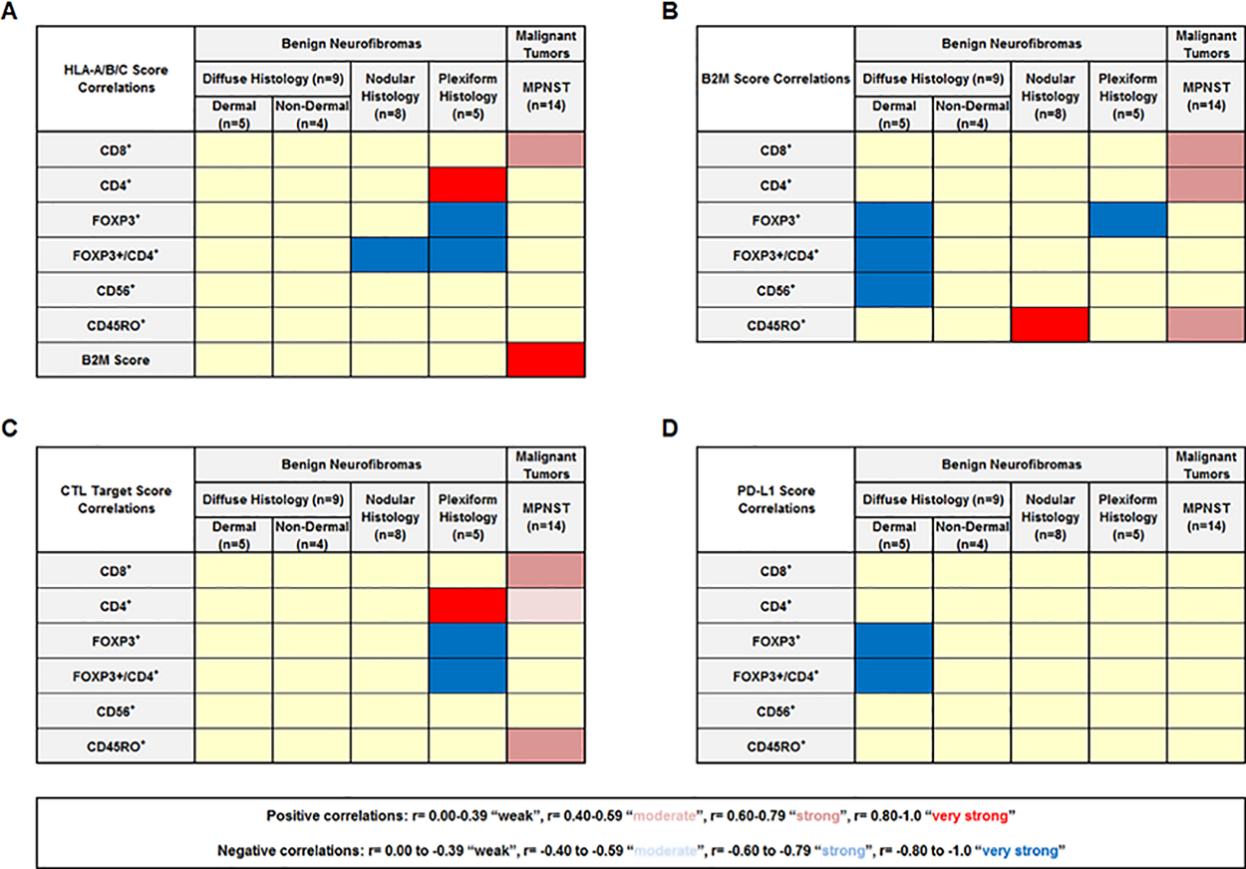
Significant correlations between *in vivo* immunohistochemical staining scores and immune cellular infiltrates **(A)** HLA score correlations; **(B)** B2M score correlations; **(C)** CTL Target Score correlations; and **(D)** PD-L1 score correlations. Number of samples per tumor subtype listed in parentheses below tumor subtype labels. Results were considered to be statistically significant when p ≤ 0.05. p value based on two-tailed t-test. r = Pearson correlation coefficient. Positive correlations are defined as: r= 0.00-0.39 “weak”, r= 0.40-0.59 “moderate”, r= 0.60-0.79 “strong”, r= 0.80-1.0 “very strong”. Negative correlations are defined as: r= 0.00 to -0.39 “weak”, r= -0.40 to -0.59 “moderate”, r= -0.60 to -0.79 “strong”, r= -0.80 to -1.0 “very strong”. Yellow shading indicates results which were not considered to be statistically significant.

We performed Spearman correlation calculations to determine whether the expression of immunologic molecules correlate with each other or with lymphocytic cellular infiltrate patterns observed in primary tumors (Figure 5 and Supplementary Table 2). The HLA-A/B/C score correlated with B2M expression for MPNST samples but not individual benign neurofibroma subtypes (Figure 5A). We found that the HLA-A/B/C score positively correlated with CD4^+^ cellular infiltrates in plexiform tumors and CD8^+^ cellular infiltrates in MPNST tumors, while negatively correlated with the FOXP3^+^/CD4^+^ cellular infiltrate ratio for nodular and plexiform tumors (Figure 5A). The expression of B2M significantly correlated with CD45RO^+^ cellular infiltrates in nodular neurofibromas and MPNST tumors and with CD4^+^ and CD8^+^ infiltrating cell counts in MPNST tumors (Figure 5B). B2M expression negatively correlated with FOXP3^+^ cellular infiltrates in plexiform and dermal diffuse tumors, as well as the FOXP3^+^/CD4^+^ cellular infiltrate ratio and CD56^+^ cellular infiltrates in dermal diffuse neurofibromas (Figure 5B). We found that the calculated CTL Target Score significantly correlated with the number of infiltrating CD8^+^, CD4^+^, and CD45RO^+^ cells in MPNST and with CD4^+^ cellular infiltrates in plexiform tumors (Figure 5C). The CTL Target Score inversely correlated with the FOXP3^+^ cell count and the FOXP3^+^/CD4^+^ cellular infiltrate ratio for plexiform neurofibromas (Figure 5C). The PD-L1 staining score showed significant inverse correlation with the amount of FOXP3^+^ cellular infiltrates and the FOXP3^+^/CD4^+^ ratio in dermal diffuse tumors (Figure 5D).

## DISCUSSION

The effective management of benign NF1-associated tumors poses a challenge for clinicians, as the therapeutic standard of complete surgical resection is often unattainable due to anticipated morbidity. Even with complete surgical resection, however, recurrence is expected in approximately 20% of cases[2], justifying the search for effective and durable therapies. Additionally, there is notable risk for transformation of plexiform tumors to malignant peripheral nerve sheath tumors (MPNST), which are relatively resistant to chemo- and radio-therapies and carry a dismal prognosis. Therapeutic modalities including chemotherapy, targeted agents against various growth factors and kinases along the RAS pathway, anti-angiogenic drugs, and radiotherapy have failed to be efficacious against these highly aggressive tumors[18], though recent reports suggest MEK inhibitors may have clinical utility [19, 20]. Though immunotherapy is a potentially attractive therapeutic strategy, as it may facilitate the reinstatement of the immune system to its original function and empower it to explicitly recognize and target tumor cells, it has been largely unexplored for NF1-associated tumors.

Immunotherapeutic modalities for cancer may be categorized by their dependence or independence on tumoral MHC Class I expression and antigen presentation [21]. Therapies utilizing CTLs, such as autologous T cell transplant, adoptively transferred T cells, immune checkpoint blockade agents, and tumor vaccines inevitably require antigenic presentation in the context of an MHC Class I molecule, with the required associated B2M allowing for appropriate conformational folding [17]. Therapeutic approaches such as chimeric antigen receptor (CAR) T cells, bispecific T cell engagers (BiTEs), and targeted cytotoxic antibodies, however, are MHC Class I-independent [21]. Other immunotherapeutic strategies, such as those which utilize natural killer (NK) cells, are more likely to succeed with the absence or low expression of MHC Class I [21].

In this report we define key immunologic characteristics of benign and malignant NF1-associated tumors. In our assessment of immunologic marker gene expression in NF1-associated samples, differences observed between tumor-associated Schwann cells and their respective tumors can be accounted for by the presence and contribution of stromal and other cells within the tumor microenvironment. Thus, despite genetic changes from normal human Schwann cells noted in the tumor-associated Schwann cells themselves, those gene levels in tumor samples likely more accurately reflect the overall immune state within the tumor microenvironment. We observed decreased HLA-A/B/C gene levels in all NF1-associated cells and tumors compared with normal human Schwann cells. These findings are consistent with previously published observations of MHC Class I down-regulation in plexiform tumors [11]. When also considering the observed low B2M gene expression in MPNST tumors, our data support the hypothesis of tumoral immune escape through reduced MHC Class I expression resulting in poor antigen presentation, indicating a potential challenge to T cell-based therapeutic strategies. Our measurement of immune checkpoint ligand gene expression revealed that dermal neurofibroma Schwann cells themselves may up-regulate PD-L1 as a means of tumoral immune escape, indicating potential for benefit from PD-1/PD-L1 pathway blockade strategies. Additionally, our observations imply that MPNST and plexiform tumors may benefit from CTLA-4 immune checkpoint blockade therapy, with the majority of the CTLA-4 expression detected likely coming from the tumoral microenvironment rather than the tumor cells themselves.

Our evaluation of immunologic marker expression by flow cytometry revealed HLA-A/B/C and B2M expression in both MPNST cell lines, with variable PD-L1 and absent CTLA-4 expression. Although the specific threshold of expression of these proteins necessary for CTL engagement is not known, it is reassuring that gene down-regulation as detected in the microarray does not necessarily indicate protein disappearance as determined by immunohistochemistry. These results parallel our gene expression analysis for the NF1-associated MPNST cells. Also correlating with our gene expression data, we did not detect significant CTLA-4 protein expression in either MPNST cell line. However, we did not measure PD-L1 and CTLA-4 expression in the tumor microenvironment by this method, and this should be taken into consideration when assessing the potential clinical utility of immune checkpoint blockade agents.

To fully evaluate tumoral immunologic marker expression in the context of the microenvironment, we performed immunohistochemical staining of tumor samples from NF1 patients. Malignant and benign tumors alike demonstrated wide ranges of staining scores within their individual subtype, indicating substantial heterogeneity among tumors of the same histologic type. Our HLA-A/B/C and B2M staining results and the associated CTL Target Score calculations suggest that plexiform and nodular benign tumors and MPNST demonstrate similar capacity to present tumoral antigens to CTLs, while diffuse histology tumors may not. Somewhat divergent from our observations using gene and flow cytometric protein analyses, both benign and malignant patient tumor samples demonstrated PD-L1 expression by immunohistochemical staining. Furthermore, we did not detect a significant difference in the overall average PD-L1 expression of MPNST versus benign tumor samples, in contrast to results recently published by Shurell et al. reporting statistically more prevalent staining of PD-L1 in MPNST samples over benign lesions [15]. All tumor histologies consistently showed broad ranges of PD-L1 staining within each group, once again demonstrating substantial tumor heterogeneity and supporting individual patient tumor sampling. It is imperative, however, to consider that CTLs additionally require engagement through antigenic presentation in the context of MHC Class I, necessitating attention to a tumor’s CTL Target Score as well as the PD-L1 score prior to the selection of checkpoint blockade immunotherapeutic approaches.

The immunoscore uses CTL (CD3^+^CD8^+^) and memory (CD45RO^+^) cellular infiltrates. While we quantified CD8^+^ and CD45RO^+^ lymphocytes within tumors, to further expand on the immunoscore and consider the probable impact of immunosuppressive regulatory T cells (T_regs_, CD4^+^FOXP3^+^) on CTL function, we additionally quantified CD4^+^ and FOXP3^+^ cells. Considering that the presence of the MHC Class I molecule serves as a negative regulator of NK cell function, we measured the number of CD56^+^ cells within the tumors. Our data suggest that MPNST tumors and plexiform and nodular neurofibromas not only display a relatively high capacity for CTL engagement (CTL Target Score) and an average ability to inhibit CTLs through PD-L1 (PD-L1 score), but also exhibit relatively high numbers of infiltrating CTLs and memory cells (inferring a favorable immunoscore) and relatively low numbers of immunosuppressive T_regs_. Taken together, these findings indicate high immunogenicity and notable potential for T cell-based immunotherapeutic approaches, though this requires validation through further studies.

Our evaluation of NF1-associated tumor samples from the same patient showed differences based on tumor histologic subtype, timing, and therapeutic intervention. Our findings suggest that this patient’s recurrent tumors may have gained mechanisms of escape from immune surveillance through the induction of immunosuppressive T_regs_. These results were independent of prior therapeutic approach, and are consistent with the observed loss of immunogenicity with tumor progression, which is common among advanced cancers [22, 23]. This patient’s case illustrates the considerable heterogeneity observed in NF1-associated tumors, even within areas of the same tumor. Interestingly, the low-grade lesion predictably displayed more immunogenicity than its counterpart high-grade lesion. Additionally, amongst the autopsy samples, the high-grade metastatic MPNST lesion found within the brain exhibited far less immunogenicity than did the high-grade MPNST sample acquired from the lung at autopsy. Differences between the immunologic microenvironments within and outside of the central nervous system may account for these findings. Taken together, our data show that the patient’s overall tumoral immunogenicity and immunoscore increased over time for each tumor subtype, without correlation to therapeutic interventions. However, the clinical course consisted of overt disease progression, with subsequent need for palliative chemo- and radio-therapeutic interventions and ultimately the patient succumbing to disease. Overall, the results from this patient case imply that immunotherapies may have been useful for this patient. Though the samples obtained 13 years after original diagnosis but prior to the patient’s death may possibly reflect the immunologic impact of the therapies received, the sample size and variable locations and histologies preclude definite conclusions.

Though our immunohistochemical scoring predicts cellular infiltrates and thus potentially the immunoscore for NF1-associated tumors, whether these measures may be useful biomarkers to predict immunotherapeutic response should be formally tested in prospective clinical trials. Our scoring potentially offers a relatively fast, easy, and inexpensive way to guide the rational selection of immunotherapeutic treatments.

Tumor heterogeneity was evident in our study, even in samples obtained from the same patient. It is therefore apparent that the score of one tumor sample is not universally predictive. Similarly, there is considerable diversity from case to case.

Though various neurofibroma subtypes have been classified, the clinical significance of these groups has been unclear for non-plexiform tumors. Our immunologic profiling of benign NF1-associated tumors reveals distinctive differences among the histologic subtypes. There were surprisingly unexpected similarities noted between the nodular and plexiform histologies, which in turn consistently mirror the trends observed in MPNST. As plexiform tumors are known to be the most likely to transform, the patterns observed here possibly indicate a more aggressive nature of nodular neurofibromas than originally thought. These three tumor subtypes display a seemingly highly immunogenic profile, in contrast to diffuse histology tumors. The observed similarities between nodular and plexiform neurofibromas and MPNST samples suggest that immunotherapies may have merit as a novel therapeutic strategy for treating these difficult tumors. Though their immunologic profiles lend to such categorization, whether these observed variances correlate with prognosis or are predictive of immunotherapeutic response will require validation with prospective studies.

## MATERIALS AND METHODS

### Microarray mining

We used a pre-clinical and clinical mRNA microarray Affymetrix database consisting of thirteen dermal neurofibromas, thirteen plexiform neurofibromas, six MPNST tumors, and thirteen MPNST cell lines and normalized their results against ten samples of normal human Schwann cells (GSE14038) to identify MHC I (HLA-A, -B, and –C), B2M, PD-L1 (CD274), and CTLA-4 gene expression [24]. We used Affymetrix GeneChip Human Genome U133 Plus 2.0 Array to measure gene expression. Data were processed using GeneSpring GX v7.3.1 (Agilent Technologies) with background correction and Robust Multi-array Average (RMA) normalization. Differential gene expression was predicted using Bioconductor/ limma package [25].

### Cell culture

The human *NF1 −/−* MPNST cell line S462TY and *NF1* ^*+*^*/*^*+*^ human sporadic MPNST STS-26T cell line have been previously described [26–28]. Cells were grown in Dulbecco’s modified Eagle’s medium (DMEM) supplemented with 20% fetal bovine serum (FBS), 100 IU/ml penicillin, and 100 μg/ml streptomycin [27]. All cells were cultured at 37°C in a humidified atmosphere containing 5% carbon dioxide. The identities of all cell lines used were confirmed by Short Tandem Repeats (STR) verification. All cell lines used in this study were found to be free of mycoplasma using MycoAlert™ Mycoplasma Detection Kit (LT07-318, Lonza Inc. Allendale, NJ).

### Flow cytometry

To assess HLA-A/B/C, B2M, PD-L1, and CTLA-4 protein expression in human MPNST cell lines, 2.5x10^5^ cells were harvested with Cell Dissociation Buffer Enzyme-Free PBS-based (Thermo Fisher Scientific, catalog number 13151014). The tumor cells were pelleted by centrifugation, resuspended in 100 μl buffer (PBS, 1% FBS, 1% EDTA), and blocked with 20% final volume of human Ig Fc blocking antibody (Miltyni Biotech, catalog number 130-059-901). Cell suspensions were stained with 1:100 dilutions of Phycoerythrin-conjugated anti-human HLA-A/B/C antibody (W6/32) (eBioscience, catalog number 12-9983-42), Phycoerythrin-conjugated anti-human β-2-microglobulin antibody (2M2) (Biolegend, catalog number 316306), Phycoerythrin -Cyanine 7-conjugated anti-human PD-L1 antibody (29E.2A3) (Biolegend, catalog number 329717), or Allophycocyanin-conjugated anti-human CTLA-4 antibody (L3D10) (Biolegend, catalog number 349907) for 30 minutes at 4°C. Following primary stain, samples were washed twice with buffer, resuspended in 1% paraformaldehyde fixative, and analyzed on a BD FACS LSR II using BD FACSDiVa software version 4.0 (BD Biosciences). Flow cytometry data analysis was performed using FlowJo version 7.6.5 (Tree Star).

### Tissue microarray construction

The Institutional Review Board at Nationwide Children’s Hospital approved this study. Thirty-six NF1-associated tumor samples consisting of nine diffuse histology benign neurofibromas (five dermal and four non-dermal samples), eight benign neurofibromas with nodular histology, five plexiform neurofibromas, and fourteen malignant peripheral nerve sheath tumors were obtained through the Nationwide Children’s Hospital Pathology Department. These samples were included onto tissue microarrays constructed from 2 mm punches of formalin-fixed paraffin-embedded tissue blocks.

### Immunohistochemical staining

All stages of the immunohistochemistry procedure were automated on the Bond III Immunohistochemistry (IHC) system (Leica Microsystems) using the Refine polymer detection system, with 3, 3’-diaminobenzidine (DAB) visualization and counterstain with hematoxylin.

An antibody to HLA Class 1 ABC (clone EMR8-5, Abcam Inc, catalog number ab70328) was used at a dilution factor of 1:6000, after on-line antigen retrieval in a citrate buffer, pH 6 (ER1, Leica Microsystems, catalog number AR9961) for 20 minutes at 100° C. β-2-Microglobulin antibody (HPA006361, rabbit polyclonal, Sigma-Aldrich, catalog number HPA006361) was used at a dilution of 1:6000 with EDTA retrieval (ER2, Leica Microsystems, catalog number AR9640) for 20 minutes at 100° C. An antibody to PD-L1 (clone B55, Sino Biological Inc., catalog number 10084-H08H) was used at a dilution factor of 1:200, after on-line antigen retrieval in an EDTA buffer, pH 9 (ER2, Leica Microsystems, catalog number AR9640) for 20 minutes at 100° C.

Antibodies to CD4 (clone SP35, Cell Marque, catalog number 104R-18) and CD8 (clone 4B11, Leica Microsystems, catalog number PA0183) were used pre-diluted, after on-line antigen retrieval in an EDTA buffer, pH 9 (ER2, Leica Microsystems, catalog number AR9640) for 20 minutes at 100° C. Antibodies to CD56 (clone 1B6, Leica Microsystems, catalog number NCL-L-CD56-1B6) and CD45RO (clone UCHL1, Leica Microsystems, catalog number PA0146) were used pre-diluted, after on-line antigen retrieval in a citrate buffer, pH 6 (ER1, Leica Microsystems, catalog number AR9961) for 20 minutes at 100° C. An antibody to FOXP3 (clone 236A/E7, Abcam Inc., catalog number ab20034) was used at a dilution factor of 1:100, after on-line antigen retrieval in an EDTA buffer, pH 9 (ER2, Leica Microsystems, catalog number AR9640) for 20 minutes at 100° C.

### Evaluations

#### Scoring of HLA-A/B/C, B2M, and PD-L1 expression in NF1-associated tumor samples by immunohistochemical staining

Immunohistochemical staining of HLA-A/B/C, B2M, and PD-L1 was scored by a single pathologist (MAA), based upon maximal staining intensity on a scale of 0 to 3 (absent = 0, weak = 1, moderate but less than normal cells = 2, or strong = 3) and percentage of tumor cells stained (range 0 to 100%), as previously described [17].

### Calculation of CTL Target Scores

For tumor samples where HLA-A/B/C and B2M expression were detected by immunohistochemical staining, CTL Target Scores were calculated as previously described [17]:

Average HLA-A/B/C score (range 0 to 300) = % positive staining × staining intensity/n. Average B2M score (range 0 to 300) = % positive staining × staining intensity/n.

CTL Target Score (range 0 to 900) = average HLA score × average B2M score/100.

### Scoring of CD4^+^, CD8^+^, CD56^+^, FOXP3^+^, and CD45RO^+^ lymphocytic cellular infiltrates

Immunohistochemical staining of CD4^+^, CD8^+^, CD56^+^, FOXP3^+^, and CD45RO^+^ cellular infiltrates was scored by the same pathologist (MAA). Counts were based upon the number of positively stained lymphocytes per square area of tumor present.

### Statistical analysis

Tumor subtype immunohistochemical staining scores and cellular infiltrates were compared using Kruskal-Wallis non-parametric testing with post-hoc pairwise comparison using the two-step method of Benjamini, Krieger, and Yekutieli to control for a false discovery rate of 0.05 using GraphPad Prism 7 software. Results were considered to be significant when adjusted p ≤ 0.05.

Correlation of immunologic marker IHC staining and calculated CTL Target Scores with tumoral immune cellular infiltrates was calculated using Pearson correlation due to expected non-Gaussian curve distribution. Sample score values were determined to positively correlate with cellular infiltrates when Pearson R = 0.00 to 0.39 (weak), 0.40 to 0.59 (moderate), 0.6 to 0.79 (strong), and 0.8 to 1.0 (very strong). Sample score values were determined to negatively correlate with cellular infiltrates when Pearson R = 0.00 to -0.39 (weak), -0.40 to -0.59 (moderate), -0.6 to -0.79 (strong), and -0.8 to -1.0 (very strong). Two-tailed t-test p values were used for all calculations. Results were considered to be statistically significant when p ≤ 0.05.

## SUPPLEMENTARY MATERIALS FIGURES AND TABLES







## Abbreviations

NF1: neurofibromatosis type 1
HLA: human leukocyte antigen
B2M: β-2-microglobulin
PD-L1: programmed death ligand 1
CTLA-4: cytotoxic T-lymphocyte antigen-4
MPNST: malignant peripheral nerve sheath tumor
CTL: cytotoxic T lymphocyte
MHC: major histocompatibility complex
MHC2TA: MHC II transactivator
TAP1: transporter associated with antigen processing
CAR: chimeric antigen receptor
BiTE: bi-specific T cell engager
NK: natural killer
RMA: Robust Multi-array Average
IHC: immunohistochemistry
DMEM: Dulbecco’s modified Eagle’s medium
FBS: fetal bovine serum
NHSC: normal human Schwann cell
dNFSC: dermal neurofibroma Schwann cell
pNFSC: plexiform neurofibroma Schwann cell
dNF: dermal neurofibroma
pNF: plexiform neurofibroma
MPNSTc: malignant peripheral nerve sheath tumor cell
MFI: mean fluorescence intensity
NF: neurofibroma
IHC: immunohistochemistry
Dx: diagnosis
R: right
L: left
yrs: years
