# Supplementary material for: Immune profiling of NF1-associated tumors reveals histologic subtype distinctions and heterogeneity: implications for immunotherapy
Source: Oncotarget. 2017 May 30;8(47):82037–48. doi: 10.18632/oncotarget.18301 (PMC5669868; doi:10.18632/oncotarget.18301)
Supplement: Supplementary file 1 [file oncotarget-08-82037-s001.pdf]

Immune profiling of NF1-associated tumors reveals histologic subtype distinctions and heterogeneity: implications for immunotherapy

SUPPLEMENTARY MATERIALS

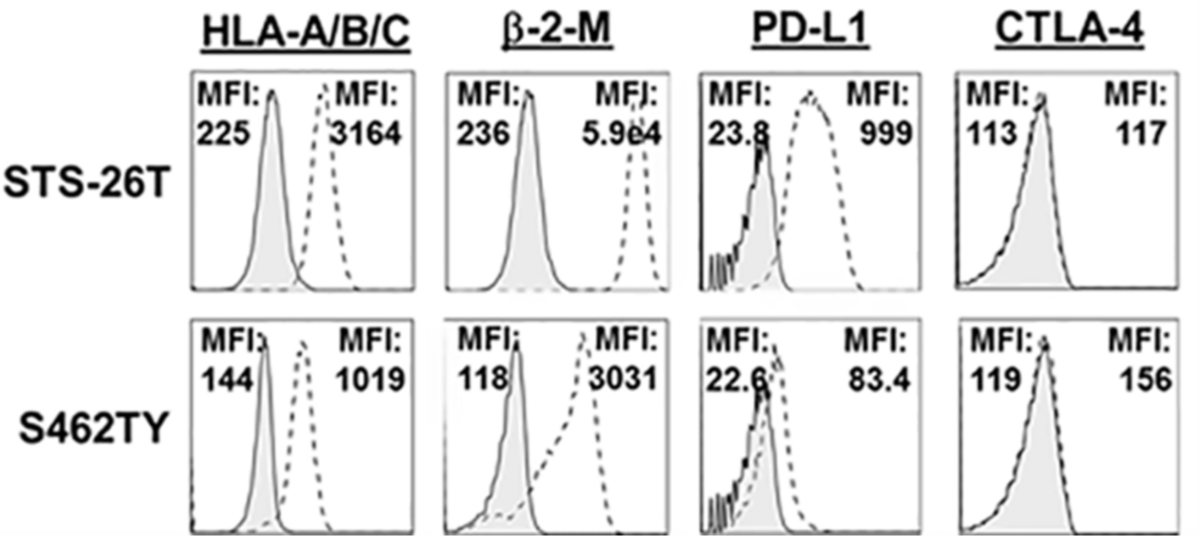

Supplementary Figure 1: *In vitro* assessment of immunologic marker expression on human MPNST cell lines STS-26T (spontaneous MPNST) and S462TY (NF1-associated MPNST) by flow cytometry. Shaded peaks represent negative control, while dashed lines represent stained samples. Data represent averages of 3 biologic replicates. MFI= mean fluorescence intensity.

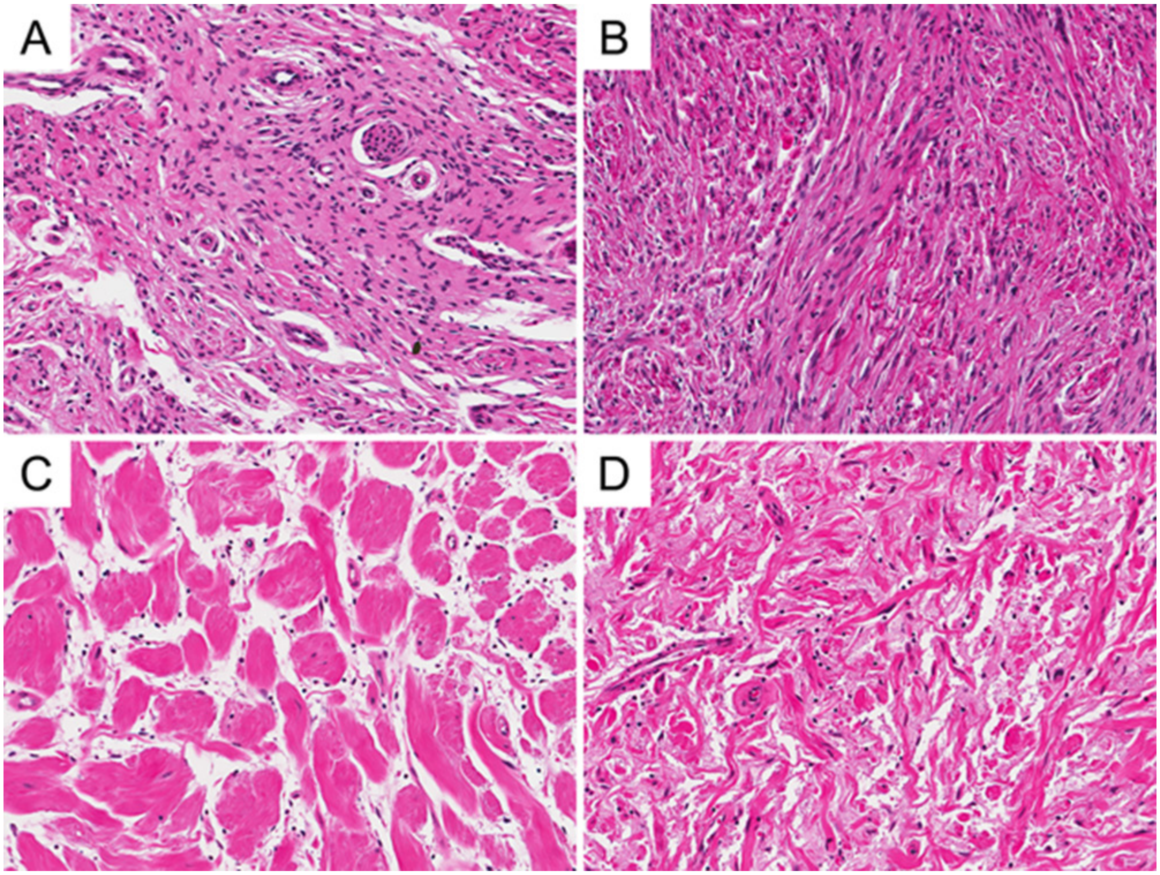

Supplementary Figure 2: Histopathologic appearance of benign neurofibroma subtypes. **(A)** Dermal diffuse histology; **(B)** non-dermal diffuse histology; **(C)** nodular histology; and **(D)** plexiform histology.

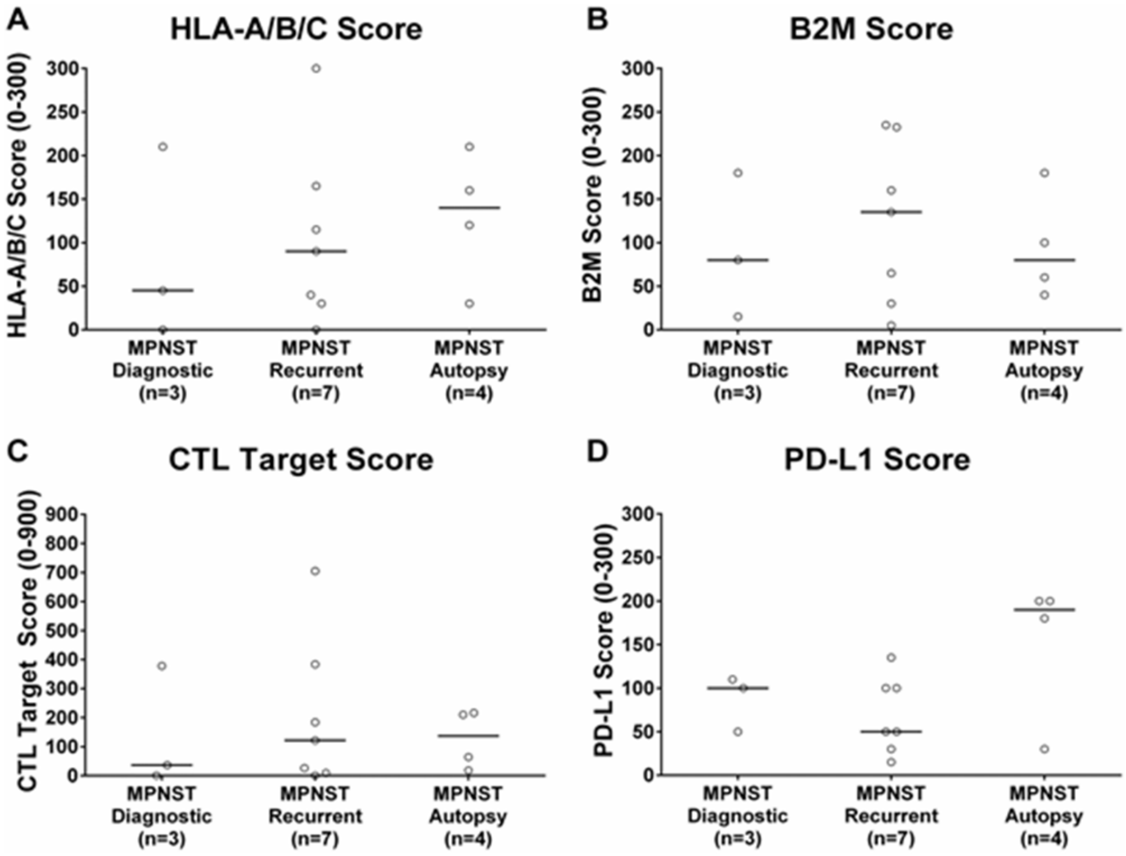

Supplementary Figure 3: *In vivo* protein expression of immunologic markers in MPNST samples by immunohistochemistry, by sample type. (A) HLA-A/B/C, range 0 to 300; **(B)** B2M, range 0 to 300; **(C)** CTL Target Scores, range 0 to 900; and **(D)** PD-L1, range 0 to 300. Each data point represents average of 3 technical replicates for that sample. Number of samples per tumor subtype listed in parentheses below tumor subtype labels. Median of all samples within tumor subtype represented.

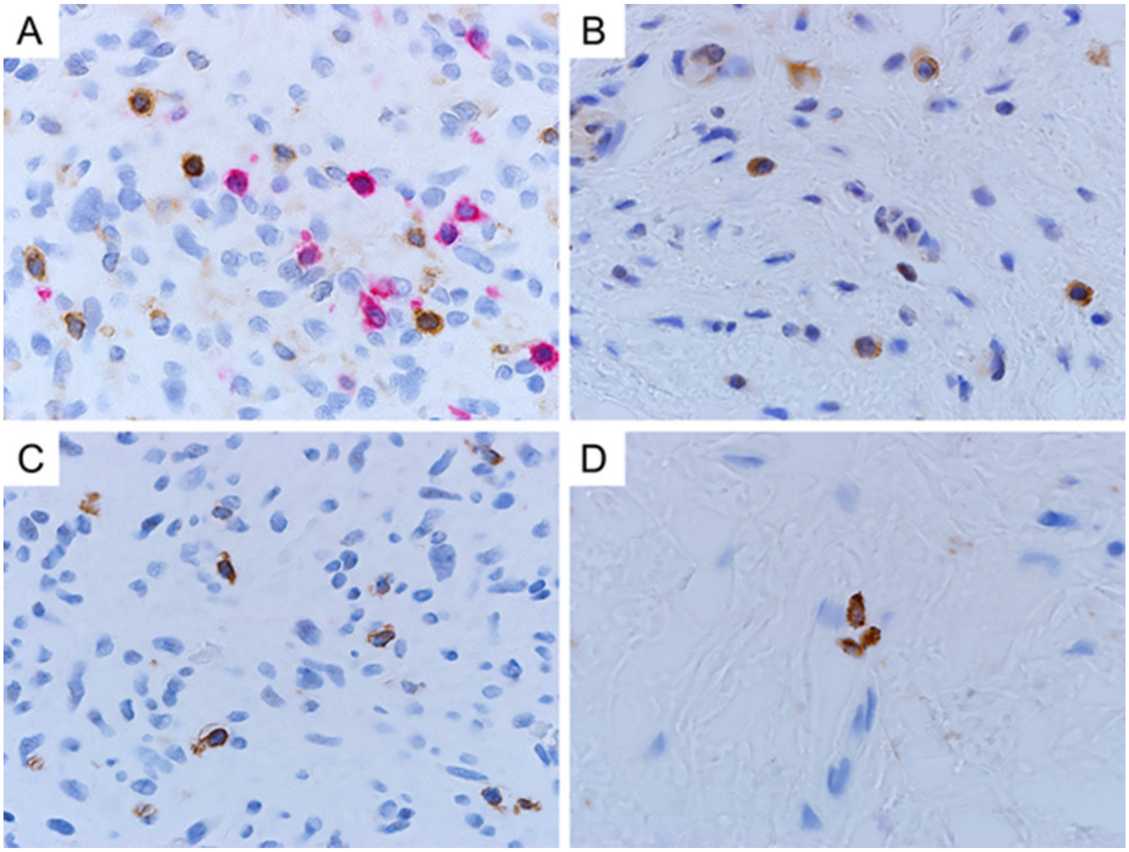

Supplementary Figure 4: Tumoral lymphocytic infiltrate staining by immunohistochemistry. (A) CD8 (red) and CD4 (brown); **(B)** FOXP3; **(C)** CD45RO; and **(D)** CD56. (100x objective.)

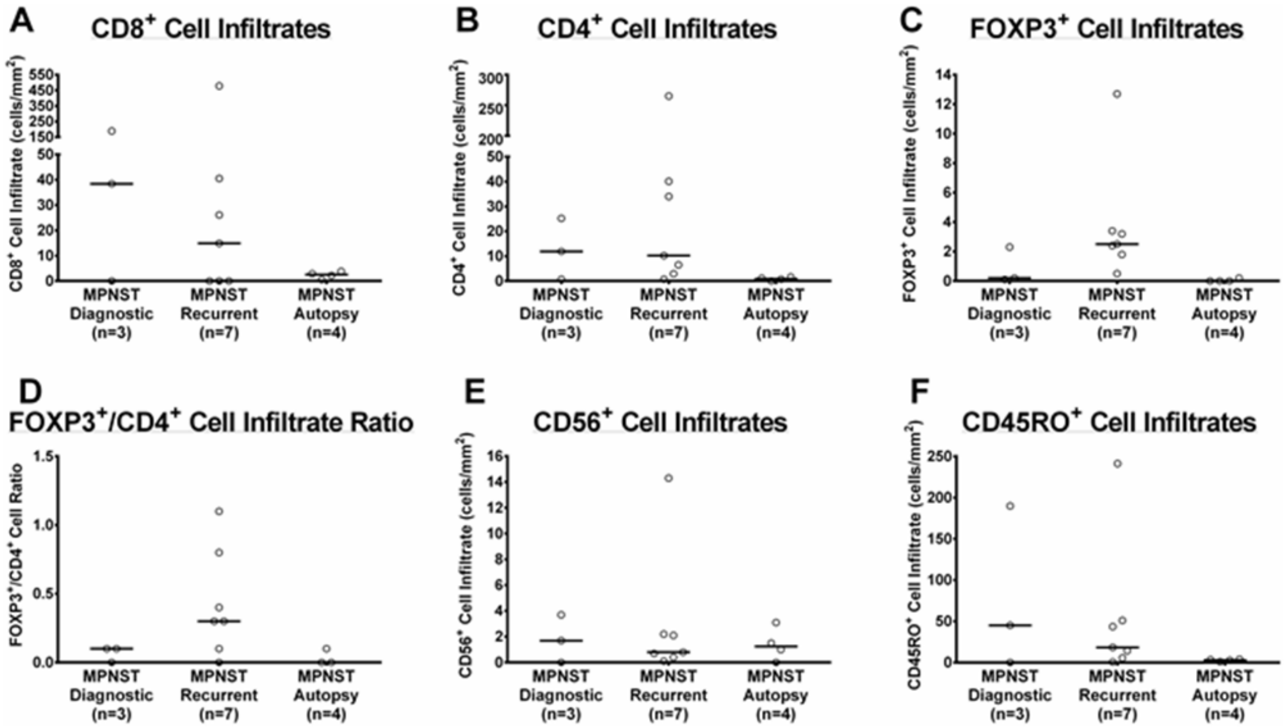

Supplementary Figure 5: *In vivo* cellular infiltrates in MPNST samples by immunohistochemistry, by sample type. **(A)** CD8$^+$ cellular infiltrates; **(B)** CD4$^+$ cellular infiltrates; **(C)** FOXP3$^+$ cellular infiltrates; **(D)** FOXP3$^+$/CD4$^+$ cellular infiltrate ratio; **(E)** CD56$^+$ cellular infiltrates; and **(F)** CD45RO$^+$ cellular infiltrates. Each data point represents average of 3 technical replicates for that sample. Number of samples per tumor subtype is listed in parentheses below tumor subtype labels. The median of all samples within tumor subtype is shown.

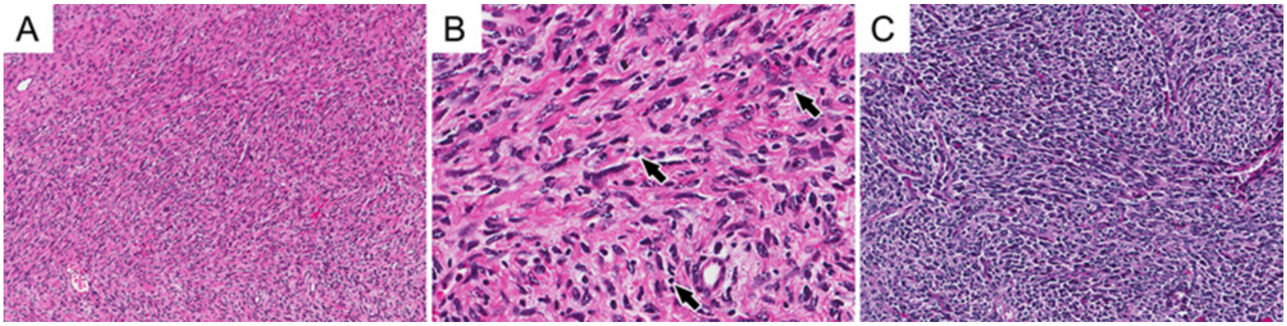

Supplementary Figure 6: Histopathologic heterogeneity observed in one MPNST sample. **(A)** Low-grade portion (H&E, 20x objective); **(B)** lymphocytic infiltrates within the low-grade portion (arrows, H&E, 40x objective); and **(C)** high-grade portion (H&E, 20x objective).

Supplementary Table 1: Demographics of NF1-associated tumors from the same patient

See Supplementary File 1

Supplementary Table 2: Significant correlations between *in vivo* IHC staining scores and cellular infiltrates

Number of samples per tumor subtype listed in parentheses below tumor subtype labels. r = Pearson correlation coefficient. p = based on two-tailed t-test. Results were considered to be statistically significant when $p \leq 0.05$.

See Supplementary File 2
